# Eco-friendly orange peel extract as corrosion resistant for carbon steel's deterioration in petroleum formation water

**DOI:** 10.1038/s41598-023-47916-w

**Published:** 2023-12-11

**Authors:** Olfat E. Elazabawy, Enass M. Attia, N. H. Shawky, Amira M. Hyba

**Affiliations:** 1 Egyptian Petroleum Research Institute, (EPRI), Nasr City, Cairo, 11727 Egypt; 2 Chemistry Department, Faculty of Science, Al-Azhar University (Girls), Nasr City, Cairo, Egypt

**Keywords:** Corrosion, Sustainability

## Abstract

The goal of the ongoing study is to determine how orange peel extract (OPE), an environmentally benign additive, affects the corrosion resistance of carbon steel in formation water (FW). The study utilized diverse techniques to investigate the effects of different peel extract concentrations, ranging from 0.5 to 2.5 percent (v/v), and concentrations of 100 to 500 ppm at room temperature (25 °C). Subsequently, the optimal concentration of 2.5 percent (v/v) was identified, and the temperature range was expanded to 25–55 °C for further examination. These techniques include dielectric spectroscopy (EIS), potentiodynamic polarisation, open circuit potential, and weight loss quantification. The inhibitory efficiency was assessed using the aforementioned techniques, and the results were further verified through the utilization of energy-dispersive radiation (EDS) and FTIR analyses. The outcomes of electrochemical testing demonstrated that orange peel extract (OPE) displayed significant effectiveness in preventing corrosion, with an inhibition rate of 90.13% when used at a concentration of 2.5% and a temperature of 25 °C. The findings suggested that orange peel extract (OPE) acts as a corrosion inhibitor with both inhibitory mechanisms. Its performance improves as the concentration of the inhibitor increases, conforming to the Langmuir adsorption isotherm model, and it adsorbed to the steel surface through physical adsorption. The findings revealed that orange peel extract (OPE) effectively served as a corrosion inhibitor for carbon steel by adsorbing its active components onto the surface of the steel. This adsorption process was primarily physical and followed the Langmuir isotherm.

## Introduction

Carbon steel is widely used in various industries due to its affordability and strong mechanical properties, particularly in construction and oil pipelines. However, corrosion of these pipelines poses a significant challenge in the oil production sector, leading to substantial economic losses. The corrosion is primarily caused by the presence of formation water in oil fields, which contains high concentrations of chloride and sulfate ions, as well as dissolved gases like carbon dioxide and hydrogen sulfide. When carbon dioxide dissolves in water, it forms carbonic acid^1–6^. Disposing of this extracted water from petroleum is inconvenient due to its high salinity and oil contamination. As a solution, the water is reinjected into oil wells to enhance crude oil recovery and increase oil production. To prevent scale deposition and corrosion in oil pipelines, chemical products with scale and corrosion-resistant properties are added to the reinjected fluid^6–8^.

Inhibitors are widely recognized as an effective solution for corrosion prevention due to their high performance, economic benefits, and widespread use in various industries. The efficiency of inhibitors depends on their physical properties and chemical structures, including polar groups, heteroatoms, aromatic rings, and pi electrons^9^. However, conventional inhibitors have drawbacks such as high toxicity, environmental degradation, and costliness, making them unsuitable for certain applications^10,11^. As a result, researchers are increasingly exploring natural products such as plants, vegetables, and fruits as alternative corrosion inhibitors. These natural products contain active biomolecules that can interact with metal surfaces, are cost-effective, and non-toxic^12–17^.

The utilization of food waste, such as fruit peels, and plant extracts as corrosion inhibitors has been extensively studied for various metals and alloys due to their reduced waste generation and abundance in nature. These waste products and extracts contain proteins, polycarboxylic acids, polysaccharides, alkaloids, and other compounds that exhibit excellent corrosion-inhibitory properties. Extracts are particularly valuable as they can be obtained at low costs using simple extraction techniques such as acidic, neutral, or basic extraction^18–21^. The concept of “environmentally friendly” or “green” corrosion inhibitors refers to compounds that are biodegradable, eco-friendly, inexpensive, and readily available. Plant extracts containing organic chemical compounds like amino acids, alkaloids, pigments, and tannins are considered green alternatives to toxic substances^22–25^.

Orange peel is a rich source of various compounds such as pectin, hesperidin, polyphenols, carotenoids, and vitamins, which contain abundant heteroatoms like oxygen (O) and nitrogen (N). These structural characteristics suggest that orange peel extract (OPE) has the potential to act as a corrosion inhibitor^17^. In a previous study by Zhang and Zhao, the inhibitory effects of orange peel extract (OPE) on the corrosion of mild steel were demonstrated in a corrosive environment containing CO_2_, H_2_S, and NaCl. The use of OPE on Q235-C steel resulted in a protection rate of 73.1%^17,26^. Wu et al. conducted research that revealed the effectiveness of orange peel extracts as a biodegradable corrosion inhibitor for magnesium alloy in a NaCl solution. Remarkably, even at a low concentration of 0.030 g/L, the extracts displayed an efficiency of 85.7%^27^. Ayodeji et al. investigated the corrosion inhibition efficiency of waste orange peels on A36 mild steel in a 1M HCl solution. The analysis indicated that the extract exhibited the highest inhibition efficiency of 94.33% when used at a concentration of 4 g/L and a temperature of 32 °C^28^.

The objective of the present research is to prepare orange peel extract and evaluate its effectiveness as an anti-corrosion agent on the surface of carbon steel in the presence of formation water, which serves as a corrosive medium. Various techniques such as weight loss measurement, potentiodynamic polarization, open circuit potential, and dielectric spectroscopy (EIS) were employed to assess the inhibition efficiency. The effectiveness of inhibition was further confirmed through energy dispersive radiation (EDS) and Fourier-transform infrared spectroscopy (FTIR) studies. Scanning electron microscopy (SEM) and energy-dispersive X-ray diffraction (EDX) were utilized to examine the morphology of the carbon steel surface and verify the formation of protective films.

## Materials and methods

### Materials

In the experimental setup, a carbon steel (CS) working electrode was utilized, which was placed inside a glass tube that was filled with Araldite epoxy. The exposed surface area of the electrode measured 0.867 cm^2^. The composition of the carbon steel, expressed as weight percentages, consisted of the following elements: Mo 0.0050, Al 0.0053, Si 0.15, P 0.0151, Ni 0.0161, Cr 0.0335, Cu 0.0398, C 0.431, with Fe comprising the remaining portion. To conduct weight loss measurements, a rectangular sample measuring 7.5 × 2 × 0.1 cm was cut from a larger sheet of mild carbon steel. An opening was made at one edge of the sample to suspend it in the corrosive medium, and it was secured in place using a plastic string. Prior to the experiment, the carbon steel samples underwent manual abrasion using emery paper, followed by washing with 100% ethanol, rinsing with double-distilled water, and drying.

The corrosive medium used in the experiment was an oiled-water solution obtained from Qarun Petroleum Company, specifically from the NED-1 water source well in Egypt (referred to as QPC). Tables 1 and 2 present the chemical composition and physical characteristics, respectively, of the formation water sample obtained from this source.

**Table 1:** The characteristic constituents of formation water solution (in ppm).

Sodium & Potassium	2205
Calcium	262
Magnesium	46
Barium	3.74
Strontium	6.5
Chlorides	4537
Sulphates	1750
Bicarbonates	378
Carbonates	0
Hydroxides	0
T.D.S	11,000

**Table 2:** The physical properties of the utilized formation water.

pH	6.88
Salinity as NaCl, ppm	7449
Total hardness, ppm	842

### Orange peel extract (OPE) preparation

The fruit underwent a cleaning process, followed by removal of the husk and drying. Subsequently, the dried husk was crushed using a mixer. In a vessel, a mixture of 50 mL ethanol and 50 mL distilled water, which had been freshly boiled and left for 30 min, was prepared. A mass of 5 g of the crushed husk was added to this mixture and intermittently mixed. After the extraction, the sample was filtered, and the resulting extracts were stored at 4 °C^29^.

The concentration of the stock solution was determined by drying a sample and calculating the remaining weight relative to the volume of the sample taken. Various extract concentrations were prepared using dilution^30^.

**Figure 1:**
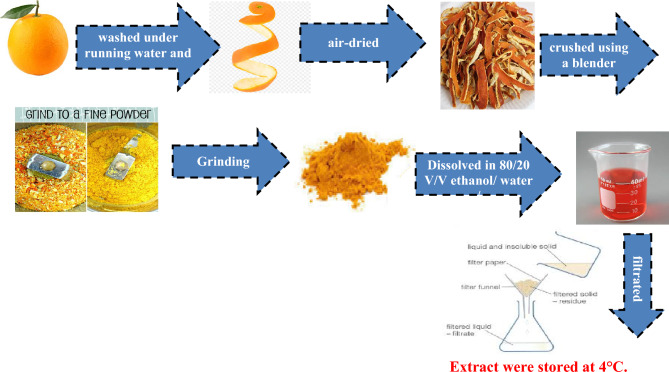
Preparation of orange peel extract (OPE).

A schematic diagram regarding the extraction process is obvious in Fig. 1.

The use of plant parts in the study complies with international, national, and/or institutional guidelines. All data generated or analyzed during this study are included in this published article.

### Methodology

#### Weight loss measurements

At the beginning of the experiment, carbon steel samples were weighed and immersed in 100 mL of formation water without any additives (referred to as the “blank” solution), as well as in the presence of various concentrations of orange peel extract (OPE) inhibitors (ranging from 0.5 to 2.5 percent (v/v) or 100 to 500 ppm of peel extract) at a temperature of 25 °C. After the specified immersion time had passed and the temperature was adjusted accordingly, the carbon steel samples were removed from the test solution and dried in a moisture-free desiccator. The samples were then reweighed, and the difference in weight between the initial and final measurements represented the weight loss.

The corrosion rate is calculated according to Eq. (1) ^31^:

1 $${C}_{R }=(\Delta W\times 534)/Atd$$

in which, ${C}_{R}$ is the corrosion rate in mpy, $\Delta W$ is the weight difference before and after immersion in the test solution in g, $t$ is the exposure time “h”, $A$ is the C-steel’s surface area “cm^2^” whereas $d$ is the C-steel's density “g/cm^3^”. Equation (2) is utilized to compute the surface coverage’s degree (θ)^32^.

2 $$ \theta = \frac{{W_{0} - {\text{W}}_{{\text{i}}} }}{{W_{0} }} $$

in which, ${W}_{i}$ and $W_{0}$ are the weight losses of C-steel in the inhibited and uninhibited solutions, respectively. Equation (3) computes the inhibition efficiency *IE* percent^33^.

3 $$IE\%=\uptheta \times 100$$

#### Electrochemical studies

A. Potentiodynamic Polarization Method

Electrochemical measurements were conducted using an electrochemical glass cell that had compartments to accommodate three electrodes. The carbon steel electrode was utilized as the working electrode (WE), while a saturated calomel electrode (SCE) and a platinum electrode (Pt) were employed as the reference electrode and auxiliary electrode, respectively. The electrode was allowed to corrode freely before each experiment, and hence its open circuit potential “OCP” was recorded for a period of up to 150 min. A permanent OCP state, which matches the working electrode's E_corr_, was then attained. The potentiodynamic polarization curves were automatically acquired by altering the electrode's potential from − 1000.00 to 0.00 mV at 2 mVs^−1^ screening rate. For each inhibitor's concentration, (0.5–2.5% (v/v) at a temperature range (25–55 °C), this technique was reiterated. On a freshly abraded electrode with a freshly made electrolyte, each experiment was carried out. To carry out the electrochemical measurements, a potentiostat (Tacussel & Radiometer PGZ 301) was used. The Voltamaster-4 software program handled this technique.

Parameters such as the potential of corrosion “*E*_corr_”, corrosion current density “*I*_corr_”, as well as Tafel’s slopes of the cathode (*β*c) along with that of the anode (*β*a) were extracted from the polarization’s curves. The inhibition's efficacy “IE percent” is computed through Eq. (4).

4 $$IE\mathrm{ \%}=\left.\left[1- \frac{{\mathrm{I}}_{\mathrm{corr}}}{{\mathrm{I}}_{\mathrm{corr}}^{^\circ }}\right.\right]\times 100$$

In which, ${I}_{\mathrm{corr}}^{^\circ }$ together with ${I}_{corr}$ = the corrosion's current densities in case of the inhibitor's absence as well as the inhibitor's presence, respectively, and the value of $\left.\left[1- \frac{{\mathrm{I}}_{\mathrm{corr}}}{{\mathrm{I}}_{\mathrm{corr}}^{^\circ }}\right.\right]$ is equivalent to the surface coverage ($\theta$)^34^.

B. Dielectric Spectroscopy “EIS” Evaluation

Within the frequency range of 100–10 mHz, keeping 10 frequency steps per decade, impedance spectra were gained at corrosion's potential after 150 min submerging time, with and without orange peel extract (OPE) inhibitor. AC signal was applied to disturb the current system employing peak-to-peak 10 mV amplitude. Zsimpwin software was utilized to interpret the data whereas the graph of Nyquist represents the EIS diagrams.

#### Surface morphology’s studies

C-steel samples were submerged for 28 days in the inhibited solution containing a specific concentration of “2.5% (v/v)” orange peel extract (OPE), as well as in the blank formation water solution, to test the surface morphological properties. Upon careful washing with distilled water 3 times by decantation, the corrosion products were then scratched with a steel scraper from carbon steel and ultimately dried up in the air.

#### SEM/ EDX analysis

For this study, a scanning electron microscope (SEM) model called Quanta 250 FEG (Field Emission Gun) was utilized, along with the energy dispersive X-ray diffraction (EDX) component. The SEM operated at an accelerating voltage of 30 kV, with a magnification range of 14X to 106,200X and a resolution of 1 nm. The equipment used was manufactured by FEI Company in the Netherlands.

#### FTIR analysis

FTIR analysis was implemented for orange peel extract as well as the corrosion products to ensure the inhibitor's adsorption on the C-steel’s surface. A Nicolet iS-10 FTIR (Thermos Fisher Scientific, USA) analysis with a resolution equal to 1 cm^–1^ was undertaken with a scanning range of four thousand to four hundred cm^−1^.

## Results and discussion

### Quantifications of weight loss

#### Inhibitor’s concentration effect

The dissolution rate of iron decreases progressively with increasing concentrations of orange peel extract (OPE), as shown in Table 3. Higher concentrations of the inhibitor lead to significant improvements in both the surface coverage (θ) and the percentage of inhibition efficacy. In particular, the inhibition efficiency reaches its highest level at approximately 92.84% when the concentration is increased from 0.5 to 2.5%. Based on this substantial enhancement in corrosion inhibition, the optimal concentration of orange peel extract for further analysis and application was chosen as 2.5%.

**Table 3:** Corrosion rates (Mpy) and inhibition efficiencies (IE %) of extracts in formation water (FW) at 25 °C.

Extract	Conc %(v/v)	${C}_{\mathrm{R}}$ (mpy)	Surface coverage (θ)	IE%
Blank	0	6.29E−08	–	–
Orange peel extract (OPE)	0.5	1.34E−08	0.7878	78.78
	1	1.25E−08	0.8011	80.11
	1.5	1.05E−08	0.8329	83.29
	2	7.85E−09	0.8753	87.53
	2.5	4.51E−09	0.9284	92.84

The improved performance of inhibition is attributed to the greater adsorption of orange peel extract (OPE) constituents on the surface of carbon steel (CS) at higher concentrations, resulting in the blocking of reaction sites^13^. This protective film formed by the inhibitor molecules effectively shields the active sites on the CS surface, leading to stronger and more adhesive protection^15^. Therefore, the concentration of orange peel extract plays a vital role in determining the degree of inhibition.

The adsorbed constituents of orange peel extract (OPE) on the CS surface inevitably lead to the blockage of reaction sites, ensuring the protection of the C-steel surface from aggressive ions present in the formation water.

#### Immersion time effect

**Figure 2:**
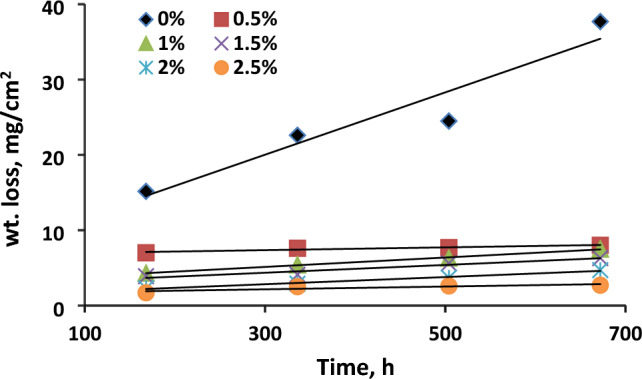
Variation of weight loss with time for corrosion of CS in formation water (FW) containing various concentrations from orange peel extract (OPE).

Figure 2 presents the weight loss variations of carbon steel (C-steel) over time when immersed in formation water alone and with different concentrations of orange peel extract (OPE) at a temperature of 25 °C. The graph clearly shows that weight loss increases as the immersion period prolongs. However, the presence of orange peel extract (OPE) leads to lower weight loss compared to the solution containing only formation water (FW). This suggests that the absence of an insoluble protective surface film formed by orange peel extract (OPE) during corrosion is responsible for the linear relationship between weight loss and time. As time passes, the stability of the protective layer formed by orange peel extract (OPE) gradually weakens, resulting in its dissolution in the corrosive solution. Therefore, the presence of an inhibitor is crucial as it initially adsorbs onto the metal surface and prevents the corrosion cycle from occurring^31^.

#### Chemical kinetics of corrosion inhibition

**Figure 3:**
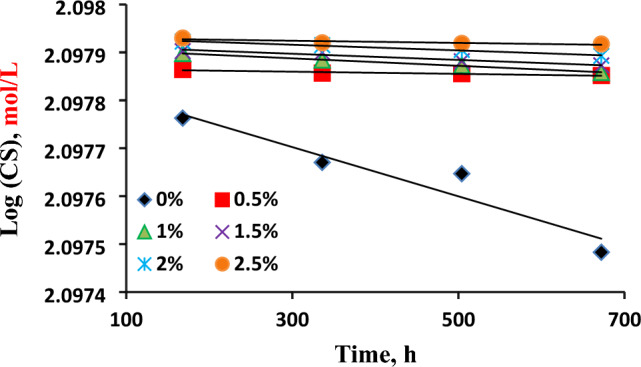
Chemical kinetic plots for the corrosion of CS in absence and presence of different concentrations of orange peel extract (OPE).

To analyze the system's kinetics, the concentration unit of corroding carbon steel (CS) is converted from mg/cm^2^ to g/L and then to molar concentrations. Assuming ‘a’ (mol/L) represents the initial concentration of carbon steel and ‘x’ (mol/L) represents the final concentration of CS after a certain time ‘t’, the corrodent concentration of CS at time ‘t’ would be (a–x) mol/L. By examining the rate law equation (Eq. 5), the order of chemical reactions can be determined. The results depicted in Fig. 3 confirm first-order kinetics, as indicated by the linear relationships with regression coefficient (R^2^) values close to unity when plotting log(ax) or log(CS) on the Y-axis against time on the X-axis.

5 $$-\mathrm{log}\left[\mathrm{corrodent}\right]=\frac{{\mathrm{K}}_{1}\mathrm{t}}{2.303}$$

where K_1_ stands for the first order rate constant, and t is the time in hours. Additionally, Eq. (6) relates the rate constant to the half-life of a first-order reaction^35^.

6 $${\mathrm{t}}_{0.5}=\frac{0.693}{{\mathrm{K}}_{1}}$$

The rate constants and half-life values obtained from the slopes of the kinetic plots are presented in Table 4.

**Table 4:** Langmuir adsorption parameters of C-steel in formation water in presence of different concentrations from orange peel extract (OPE) using weight loss method at 25 °C.

${K}_{ads}$(mol^−1^)	Slope	${\Delta G}_{ads}^{o}$(kJ/mol)	R^2^
5.36	1.0306	− 13.874	0.9938

The findings indicate that the addition of orange peel extract (OPE) extends the half-life of carbon steel (CS) compared to the blank solution. However, it is observed that the rate constant (K) of CS in the presence of orange peel extract (OPE) is lower than the rate constant of the blank solution. This demonstrates that the inhibitors, in this case, orange peel extract, effectively increase the longevity of carbon steel (CS) in the formation water (FW).

#### Adsorption isotherm

**Figure 4:**
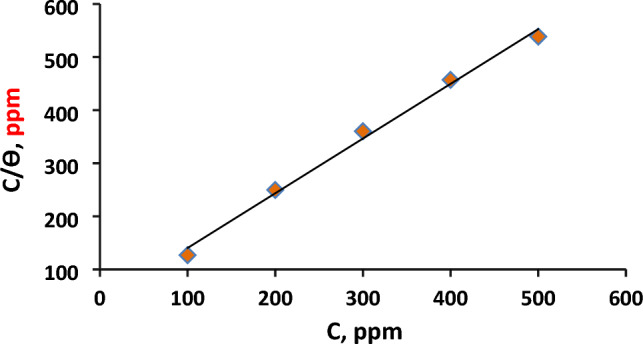
Langmuir adsorption isotherm for carbon steel in formation water containing different concentrations of orange peel extract (OPE) at 25 °C.

To further examine the adsorbing effect of the extract on the C-steel’s surface, Langmuir's adsorption isotherm is utilized by using the subsequent equation ^36^:

7 $$ {\raise0.7ex\hbox{$C$} \!\mathord{\left/ {\vphantom {C \theta }}\right.\kern-0pt} \!\lower0.7ex\hbox{$\theta $}} = {\raise0.7ex\hbox{$1$} \!\mathord{\left/ {\vphantom {1 {K_{ads} }}}\right.\kern-0pt} \!\lower0.7ex\hbox{${K_{ads} }$}} + C $$

which, *θ* = the surface coverage, *C* = the extract’s concentration in (v/v %), whereas ${K}_{ads}$ = the adsorption's equilibrium constant. *K*_ads_ can be obtained from the reciprocal of the isotherm line’s intercept.

$${\raise0.7ex\hbox{$C$} \!\mathord{\left/ {\vphantom {C \theta }}\right.\kern-0pt} \!\lower0.7ex\hbox{$\theta $}}$$ is plotted against $$C$$ as lucid in Fig. 4 and then the data of Langmuir's parameters were gathered in Table 5.

**Table 5:** Potentiodynamic parameters for CS in formation water (FW) in absence and presence of various concentrations of orange peel extract (OPE) at 25 °C.

Conc., %(v/v)	${\mathrm{E}}_{\mathrm{corr}}$ (mV/SCE)	${\mathrm{I}}_{\mathrm{corr}}$ (mA/cm^2^)	βa (mV/dec)	− βc (mV/dec)	θ	IE%	${\mathrm{C}}_{\mathrm{R}}$, (mpy)
0	− 687	41.4	123	289	–	–	251
0.5	− 693	16.3	130	189	0.6063	60.63	191
1	− 679	15.1	100	188	0.6339	63.39	177
1.5	− 687	10.7	123	150	0.7410	74.10	126
2	− 676	8.5	135	160	0.7954	79.54	99
2.5	− 693	4.0	123	155	0.9013	90.13	53

A linear relationship with an R^2^ value close to one indicates a good fit of the data to the Langmuir equation, confirming that the orange peel extract (OPE) exhibits adsorption activity on the surface of C-steel. This suggests that the active components of the extract occupy sites on the steel's surface, consistent with previous studies^22^. The Langmuir isotherm assumes that the adsorption surface is covered by a monolayer and that there is no interaction between the adsorbed molecules. As a result, the adsorption sites become saturated, and further adsorption is not possible^29^. Using Eq. (8), the standard free energy of adsorption, ${\Delta G^\circ }_{ads}$, can be calculated^37^ and is compiled in Table 5.

8 $${\Delta G^\circ }_{ads} = -R \times T ln\left({C}_{w} \times {K}_{ads}\right)$$

Here, R represents the gas constant, C_w_ is the water concentration in the solution (55.5 mol/L), and T is the absolute temperature^22^. According to thermodynamic relationships, the adsorption reaction occurs when the free energy is negative. It is worth noting that physical adsorption releases less heat compared to chemical adsorption. Throughout this research, the negative ${\Delta G^\circ }_{ads}$ value indicates the spontaneous adsorption of the extract onto the surface of the C-steel, highlighting the strong interactions between the metal's surface and the inhibitor molecules. The ${\Delta G^\circ }_{ads}$ value, which is lower than − 20 kJ/mol, demonstrates the physisorption mechanism of orange peel extract (OPE) on C-steel in the corrosive formation water at a temperature of 25 °C^30^.

### Open circuit potential assessment (OCP)

**Figure 5:**
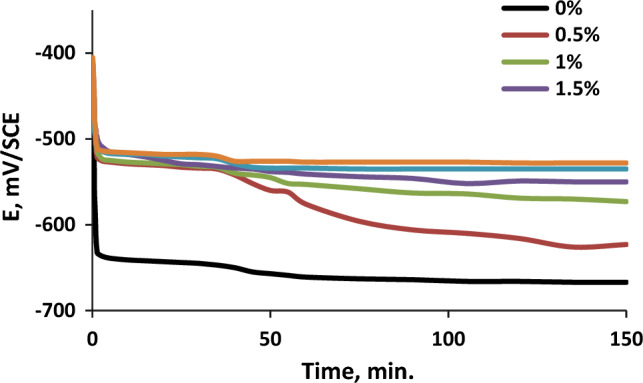
The variation of the open-circuit potential (OCP), for C steel electrode in different concentrations of orange peel extract (OPE), with time.

Figure 5 shows the OCP changes of C-steel in formation water over time. Two important observations are evident. Firstly, both free and inhibited solutions experience a gradual shift towards more negative electrode potentials during immersion, indicating the dissolution of the initial oxide film. Secondly, the presence of the orange peel extract (OPE) inhibitor causes a more positive OCP compared to free formation water. Increasing the inhibitor concentration further shifts the steady-state potential in a positive direction^38^. This demonstrates the effective inhibition of OPE through the adsorption of its constituents on the C-steel surface, forming a protective layer against corrosion^39^.

### Potentiodynamic polarization measurements

#### Inhibitor's concentration

**Figure 6:**
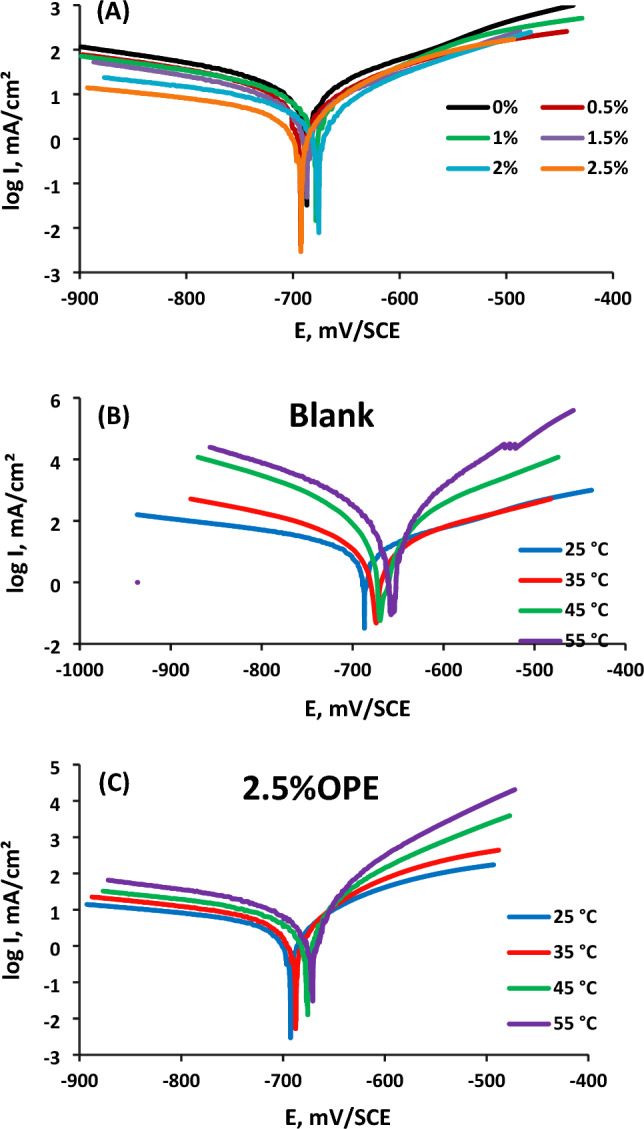
Potentiodynamic polarization plots of C-steel in formation water (FW) (**A**) in absence and presence of various concentrations of orange peel extract (OPE) at 25 °C, (**B**) at temperature range (25–55 °C) and (**C**) in presence of 2.5% orange peel extract (OPE) at temperature range (25–55 °C).

Figure 6 in the study presents polarization curves depicting the changes in anodic and cathodic electrochemical polarization of carbon steel under different conditions: without orange peel extract (OPE) in formation water and with OPE at various concentrations (25 °C). Parameters such as corrosion potential (E_corr_), corrosion current density (I_corr_), and Tafel slopes (β_c_ and β_a_) were extracted from the curves and listed in Table 6. Surface coverage (θ) and inhibition efficacy (IE percent) were also calculated and included in the table.

**Table 6:** Potentiodynamic parameters for C-steel in formation water (FW) in absence and presence of 2.5% peel extract at temperature range (25–55 °C).

	Temp. (°C)	${\mathrm{E}}_{\mathrm{corr}}$ (mV/SCE)	${\mathrm{I}}_{\mathrm{corr}}$ (mA/cm^2^)	Βa (mV/dec)	− βc (mV/dec)	${\mathrm{C}}_{\mathrm{R}}$ (mpy)	θ	IE%
Blank	25	− 687	41.4	123	289	251	–	–
	35	− 675	44.5	100	161	270	–	–
	45	− 673	79.4	124	129	360	–	–
	55	− 660	97.7	124	250	390	–	–
Orange Peel Extract (OPE)	25	− 693	4.5	123	155	53	0.9013	90.13
	35	− 695	5.6	144	86	64	0.8804	88.04
	45	− 690	7.5	156	72	81	0.8799	87.99
	55	− 682	12.6	195	42	140	0.8686	86.86

Table 6 reveals that higher OPE concentrations lead to increased inhibition efficacy percentages and decreased corrosion current density. This indicates that higher concentrations of OPE result in more inhibitor accumulation on the steel surface through adsorption, effectively inhibiting corrosion^40^. The most effective inhibition is observed at a concentration of 2.5 percent OPE.

The corrosion potential (E_corr_) remains relatively constant with varying inhibitor concentrations, suggesting that OPE acts as a mixed-type inhibitor, affecting both metal dissolution and hydrogen production. If the difference in E_corr_ between inhibited and uninhibited conditions is less than 85 mV, the inhibitor is considered to be of mixed type^41^.

The decrease in both anodic and cathodic current densities in the presence of OPE indicates its inhibitory action. This decrease becomes more pronounced at higher OPE concentrations. The adsorption of OPE components on the steel surface protects it from the corrosive medium, reducing both hydrogen evolution and iron metal deterioration^42^.

In the presence of orange peel extract (OPE), the kinetic of C-steel oxidation is minified slightly, as noticed conclusively in Fig. 6A through the anodic branch. In the harsh environment of formation water (FW), the anti-corrosion properties of OPE on C-steel are significant. The reduction in the corrosion rate of C-steel can be attributed to the suppression of both cathodic hydrogen reduction and anodic oxidation.

Additionally, the anodic Tafel slope (βa) remains unchanged with OPE, indicating that OPE's adsorption does not significantly affect the metal dissolution reaction. However, the cathodic Tafel slope (βc) increases significantly with OPE, indicating greater inhibition of the cathodic process (hydrogen evolution)^42^.

Overall, the addition of OPE results in a significant reduction in corrosion rate, particularly in the cathodic branch. This indicates that both anodic dissolution and cathodic hydrogen evolution are slowed down by OPE, with a stronger suppression of the cathodic reaction^43^.

OPE acts as a mixed inhibitor, primarily exerting a cathodic inhibitory effect in the formation water. The inhibitory activity is influenced by the presence of anions in the water, which facilitate the adsorption of inhibitor species on cathodic sites, reducing the rate of hydrogen gas evolution^11^.

#### Temperature effect

Figure 6B and C in this study illustrate the impact of temperature (ranging from 25 to 55 °C) on carbon steel (C-steel) corrosion in formation water (FW), both in the absence and presence of 2.5 percent orange peel extract (OPE). The corresponding polarization parameters were determined from the polarization curves obtained at various temperatures and compiled in Table 7.

**Table 7:** Activation thermodynamic parameters of CS in absence and presence of orange peel extract (OPE) using potentiodynamic polarization method.

	${E}_{a}^{*}$ kJ/mol	${\Delta H}^{*}$ (kJ/mol)	${\Delta S}^{*}$ J/(mol K)	− Δ G* (kJ/ mol)			
				25 °C	35 °C	45 °C	55 °C
Blank	13.12	10.52	− 163.93	59.37	61.01	62.65	64.29
Orange Peel Extract (OPE)	25.63	23.03	− 135.40	63.4	64.73	66.09	67.44

The results demonstrate the highly effective inhibition of orange peel extract (OPE) in mitigating C-steel corrosion in the harsh environment of formation water (FW) across the temperature range studied.

The data reveals a notable increase in corrosion current densities (I_corr_) as the temperature rises, and this effect is further enhanced in the absence of inhibitors. The corrosion rate of C-steel increases with temperature in both uninhibited and inhibited formation water (FW). Conversely, the inhibition efficiency (IE) of C-steel decreases with increasing temperature. This can be attributed to the potential desorption of orange peel extract (OPE) from the surface of C-steel at higher temperatures^44^.

As temperature increases, inhibitor molecules diffuse from the C-steel surface into the solution, potentially exposing more C-steel surface area to the corrosive medium. This leads to a decrease in the inhibitory efficiency (IE) percentage. In the presence of inhibitors, there is a balance between adsorption and desorption processes^45^.

The decrease in adsorption intensity at higher temperatures can be explained by a reduction in the adsorption capacity, assuming a physical adsorption mechanism. The increased temperature also enhances C-steel dissolution, which hampers the adsorption of orange peel extract (OPE) on the metal surface^44^.

The decline in the protective properties of orange peel extract (OPE) with temperature increase has two implications. Firstly, the adsorption–desorption balance shifts towards desorption, reducing the intensity of adsorption. Secondly, higher temperatures cause increased corrosion, resulting in a rougher surface for the steel. These findings suggest that orange peel extract (OPE) exhibits physical adsorption characteristics on the C-steel surface^46^.

#### Kinetic and thermodynamic corrosion parameters using potentiodynamic polarization method

**Figure 7:**
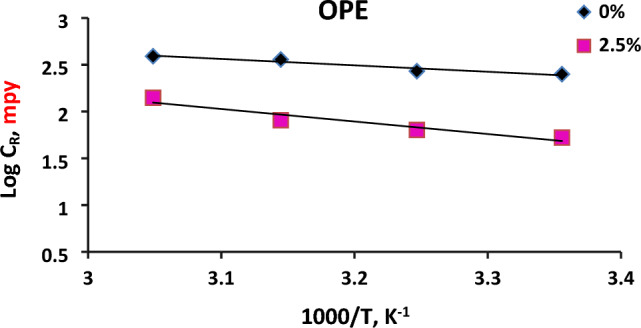
Arrhenius plots for CS in absence and presence of 2.5% orange peel extract (OPE) using potentiodynamic polarization method.

Figure 7 demonstrates the relationship between the logarithms of corrosion rate “*C*_R_” plotted versus the reciprocal of temperature “1/*T*”, for C-steel in the aggressive formation water (FW) alone as well as in the inhibited solution containing a certain concentration from orange peel extract (OPE) (2.5%). The activation energies ${E}_{a}^{*}$ were then extracted from the slope of the curve using the Arrhenius equation and subsequently listed in Table 8.

9 $$ \ln C_{R} = - E_{a}^{*} /(RT) + \ln A $$

**Table 8:** Langmuir adsorption isotherm parameters for the extract adsorbed on CS surface in formation water (FW) solution using potentiodynamic method at 25 °C.

Extract	${K}_{\mathrm{ads}}$ (L/ mol^)^	Slope	${\Delta G}_{ads}^{o}$ (kJ/mol)	R^2^	${\Delta H}_{ads}^{o}$ (kJ/mol)	${\Delta S}_{ads}^{o}$ (kJ/(mol K))
Orange peel extract (OPE)	2.03	0.967	− 11.71	0.971	− 12.31	2.013

In which, *C*_R_ = corrosion rate; A = pre-exponential factor; *E*_a_^*^ = activation energy; *R* = gas constant, and *T* = absolute temperature.

The presence of orange peel extract (OPE) resulted in a higher activation energy value compared to its absence, indicating that the extract had a lower inhibition efficiency at higher temperatures. The significant difference in activation energy values between the presence of 2.5% orange peel extract (OPE) and the blank formation water (FW) indicated that the extracts effectively delayed the corrosion of carbon steel (CS) in the formation water (FW) solution^47^.

The increase in activation energy (${E}_{a}^{*}$) in the presence of the extracts suggested that the addition of orange peel extract (OPE) to the formation water (FW) solution significantly raised the energy barrier for corrosion. This also indicated a decrease in protection efficiency as the temperature increased. The positive ${E}_{a}^{*}$ values were observed for both inhibited and uninhibited solutions (Table 8). Furthermore, in the presence of orange peel extract (OPE), the value of ${E}_{a}^{*}$ was close to 25 kJ/mol, indicating that the corrosion process was primarily controlled by surface reaction^48^. These findings indicated that the inhibitor molecules of orange peel extract (OPE) were mainly bound to the surface of CS through a physico-sorption mechanism.

The enthalpy change (${\Delta H}^{*})$ along with the entropy (${\Delta S}^{*})$ of activation for the corrosion process are computed through Eq. (10).

10 $$\mathrm{log}{C}_{R}/T= \mathrm{log}\left(R/Nh\right)+\left({\Delta S}^{*}/2.303R\right)-\left({\Delta H}^{*}/2.303RT\right)$$

In which: Planck’s constant “$h$” = 6.23 × 10^−34^ J.s., whereas, Avogadro’s number “$N$” = 6.022 × 10^23^ mol^−1^.

**Figure 8:**
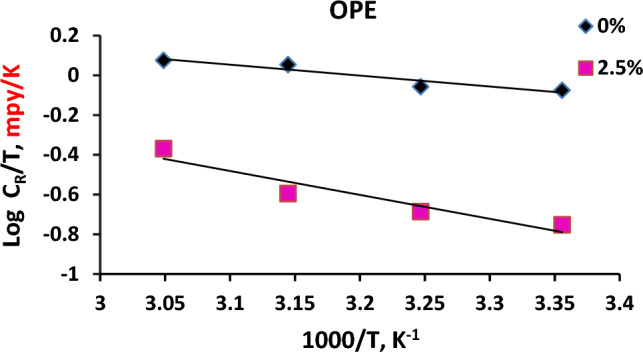
Transition state plots for CS in absence and presence of 2.5% orange peel extract (OPE) using potentiodynamic polarization method.

Upon drawing $\mathrm{log}{C}_{R}/T$ opposed to $1/T$ in Fig. 8, a straight line is acquired with a slope equal to $\left(-{\Delta \mathrm{H}}^{*} /2.303R\right)$ whereas the intercept is equal to $[\mathrm{log}\left(R/Nh\right)+\left({\Delta S}^{*}/2.303R\right)]$. Consequently, ${\Delta H}^{*}\text{as well as }{\Delta S}^{*}$ were acquired and subsequently, tabulated in Table 8.

The positive enthalpic activation energy (ΔH*) values suggest that the dissolution of the metal is endothermic. In the case of CS dissolution in formation water (FW) with orange peel extract (OPE), the ΔH* value is significantly higher (23.03 kJ/mol) compared to the blank formation water (10.52 kJ/mol). This indicates a slower rate of CS surface dissolution in the presence of orange peel extract (OPE)^49^. Based on Table 8, the enthalpy value increases when orange peel extract (OPE) is present, indicating improved effectiveness in protecting against dissolution compared to the uninhibited solution^50^.

The negative ∆S* indicates a decrease in entropy at the transition state, suggesting an associative mechanism where reactants form a complex transition state^51^. Comparing the ∆S* values of the inhibited and uninhibited formation water solutions, the inhibited solution exhibited a higher ∆S* value (-135.40 J mol^−1^ K^−1^) compared to the uninhibited solution (− 163.93 J mol^−1^ K^−1^). This slight increase in disorder after the reactants transformed into the activated complex upon the addition of orange peel extract (OPE). The adsorption of OPE inhibitor molecules from the formation water can be seen as a quasi-substitution process between OPE in the aqueous phase and water molecules on the electrode surface, resulting in an increase in solvent entropy^52^. However, in both the presence and absence of the inhibitor, the activation entropy remained high and negative, indicating that the rate-determining step involved an association rather than a dissociation step, and there was a decrease in disorder from reactants to the activated complex^53^.

The free activation energy, ΔG* is calculated at each temperature using the following thermodynamic relation:

11 $${\Delta G }^{*}= {\Delta H }^{*} - T{\Delta S }^{*}$$

The free activation energy (ΔG*) was determined at each temperature using Eq. (11) and presented in Table 9. These values are positive and slightly higher compared to the blank values. The rise in temperature enhanced the spontaneity of the corrosion process, indicating that the activated complexes became more soluble at higher temperatures. However, the increase in ΔG* with temperature indicated that the activated compound was less stable, and its formation became slightly less likely as the temperature increased. Consequently, a greater number of corrosion species entered an activated state with a less stable structure, potentially contributing to the escalation of corrosion rate with rising temperature^52^.

**Table 9:** Percentage of atomic contents of elements (Mass %) obtained from EDX spectra.

Element	Polished CS	CS in Formation Water (FW) for 1 month	CS in Formation Water (FW) and Orange Peel Extract (OPE) for 1 month
N	–	–	1.12
C	–	1.95	–
O	–	24.19	24.93
Na	–	1.95	–
Mg	–	1.08	–
Si	–	4.82	–
S	–	0.65	3.14
Cl	–	0.38	–
Ca	–	10.16	–
Fe	100	54.81	70.8
Total	100	100	100

#### Adsorption isotherm and thermodynamic adsorption parameters using potentiodynamic polarization method

**Figure 9:**
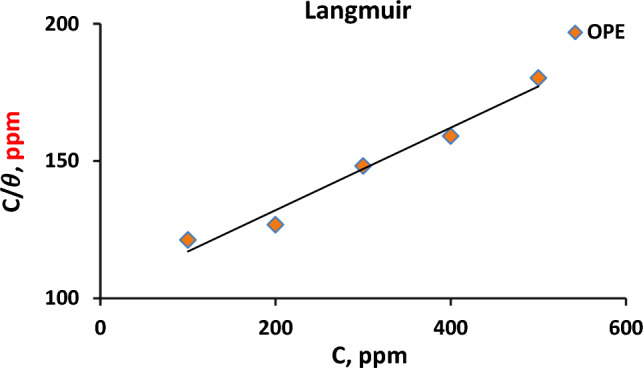
Langmuir adsorption isotherm for orange peel extract (OPE) extract adsorbed on CS surface in formation water (FW) solution using potentiodynamic method.

Thermodynamic adsorption parameters and isotherm models are crucial for understanding the mechanism of the orange peel extract (OPE) adsorption process on the submerged electrode^9^. Graphically, the degrees of surface coverage (θ) for various orange peel extract (OPE) concentrations were examined by fitting to various isotherms. Figure 9 shows that extracts follow the Langmuir adsorption isotherm, through drawing *C/θ* against *C* since $\left.\left[1- \frac{{\mathrm{I}}_{\mathrm{corr}}}{{\mathrm{I}}_{\mathrm{corr}}^{^\circ }}\right.\right]$ is equivalent to the surface coverage ($\theta$)^54^. This suggests that the cathodic and anodic regions are both covered by orange peel extract (OPE) inhibitor. According to the Langmuir isotherm, the inhibitor molecules form a single layer without interacting with nearby molecules^29^.

The values of K_ads_, which are connected to the standard free energy of adsorption through Eq. (8) ^37^, are relatively small (Table 9). This suggests that the interaction between the adsorbed extract molecules and the CS surface is of a physical nature. This is further supported by the extract's lower negative value of ∆G^o^_ads_, indicating that the orange peel extract (OPE) molecules adsorb spontaneously and firmly attach to the steel electrode surface. In the literature, values of ∆G^o^_ads_ up to 20 kJ/mol are typically associated with physical adsorption, while values above 40 kJ/mol are indicative of a chemical adsorption mechanism^54^. Since the free energy of adsorption in this case is less than 20 kJ/mol, the adsorption process of orange peel extract (OPE) occurred through physical adsorption, primarily driven by electrostatic interactions between the charged metal and the extract molecules.

The Gibbs–Helmholtz equation can be used to compute the enthalpy of adsorption (Δ*H*^*o*^_ads_) (Eq. 12)^55^.

12 $$\frac{\Delta {{G}^{o}}_{ads}}{T}= \frac{\Delta {{H}^{o}}_{ads}}{T}+{ K}_{ads}$$

Thereafter, the entropy of adsorption (**∆*S***^***o***^_**ads**_), can be computed using the following thermodynamic basic equation^56^

13 $$ \Delta S^{{\text{o}}}_{{{\text{ads}} }} = \, - \, \left( {\Delta G^{{\text{o}}}_{{{\text{ads}}}} - \, \Delta H^{{\text{o}}}_{{{\text{ads}}}} } \right) \, /T $$

The computed enthalpy and entropy of adsorption are compiled in Table 9. The negative value of adsorption enthalpy (∆H^o^_ads_) indicates that the adsorption of the orange peel extract (OPE) inhibitor is an exothermic process. An adsorption enthalpy value below 40 kJ/mol suggests physisorption, while a value reaching 100 kJ/mol indicates chemisorption^56^. Based on the ∆H^o^_ads_ value, the adsorption process of the inhibitor on the carbon steel is physisorption^57^.

The negative value of ΔS^o^_ads_ implies a decrease in system disorder after the adsorption of the orange peel extract (OPE) on the metal surface. This reduction in randomness confirms the formation of a protective OPE film on the metal surface^49^. Adsorption leads to a decrease in entropy as the molecules that were freely moving in the bulk solution become immobilized on the metal surface^58^.

In summary, the inhibitive properties of the OPE extract can be attributed to its ability to adsorb onto the metal surface, with the polar group playing a crucial role in the adsorption process. The resulting adsorbed layer acts as a barrier between the metal and the corrosive environment. The effectiveness of inhibition depends on the mechanical, structural, and chemical properties of the adsorption layer under specific conditions^59^.

### Characterization of peel extracts

**Figure 10:**
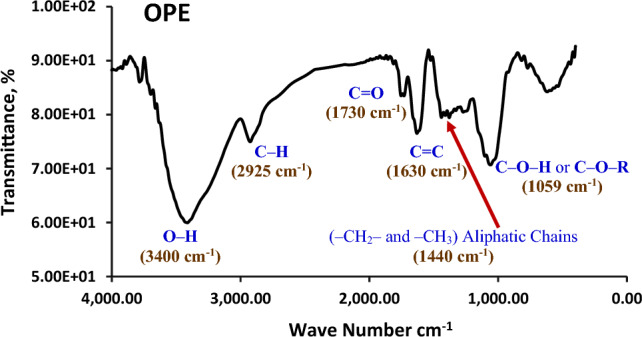
IR spectrum of orange peel extract (OPE).

The FTIR spectrum of the dried orange peel, depicted in Fig. 10, reveals valuable insights. In addition to lignin, characteristic bands corresponding to cellulose are observed. The high-energy region showcases a dominant band resulting from the combination of numerous OH groups found in lignin and carbohydrates. Notably, the intense band at 1059 cm^−1^ signifies the presence of C–O–H or C–O–R connections, specifically alcohols or esters. Furthermore, the distinct band at 2925 cm^−1^ indicates C–H stretching vibration and bending vibration around 1440 cm^−1^, originating from aliphatic chains (–CH_2_– and –CH_3_) that form the fundamental structure of this lignocellulosic material. At 1730 cm^−1^, the signal can be attributed to the presence of carbonyl groups, such as esters. Lastly, the band around 1630 cm^−1^ is likely associated with (C=C) aliphatic and/or aromatic compounds^60^. These findings shed light on the molecular composition of the orange peel.

### SEM and EDX Examination

**Figure 11:**
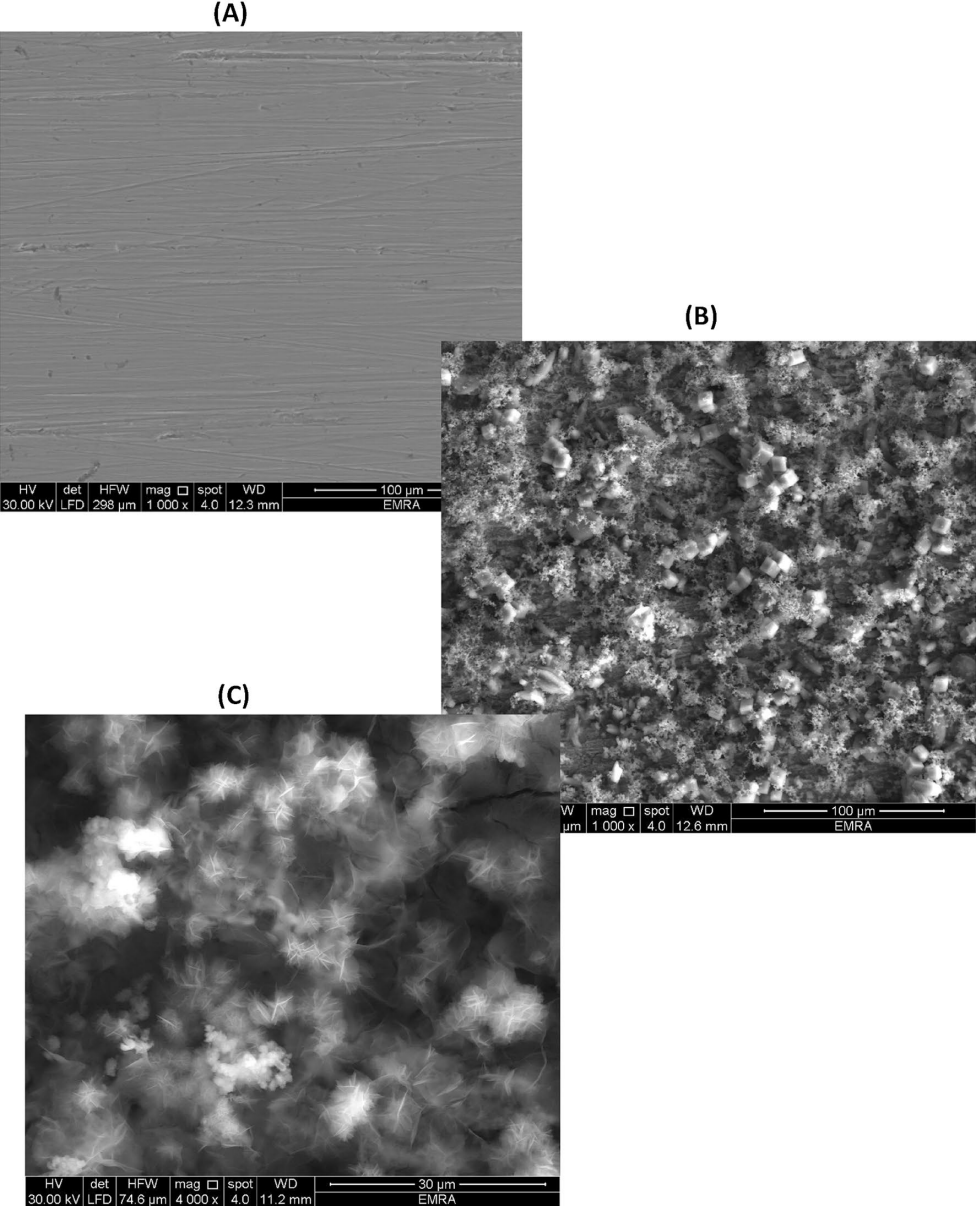
SEM micrograph for C steel coupons: (**A**) Before treatment with formation water, (**B**) in formation water and (**C**) in 2.5% of orange peel extract (OPE).

**Figure 12:**
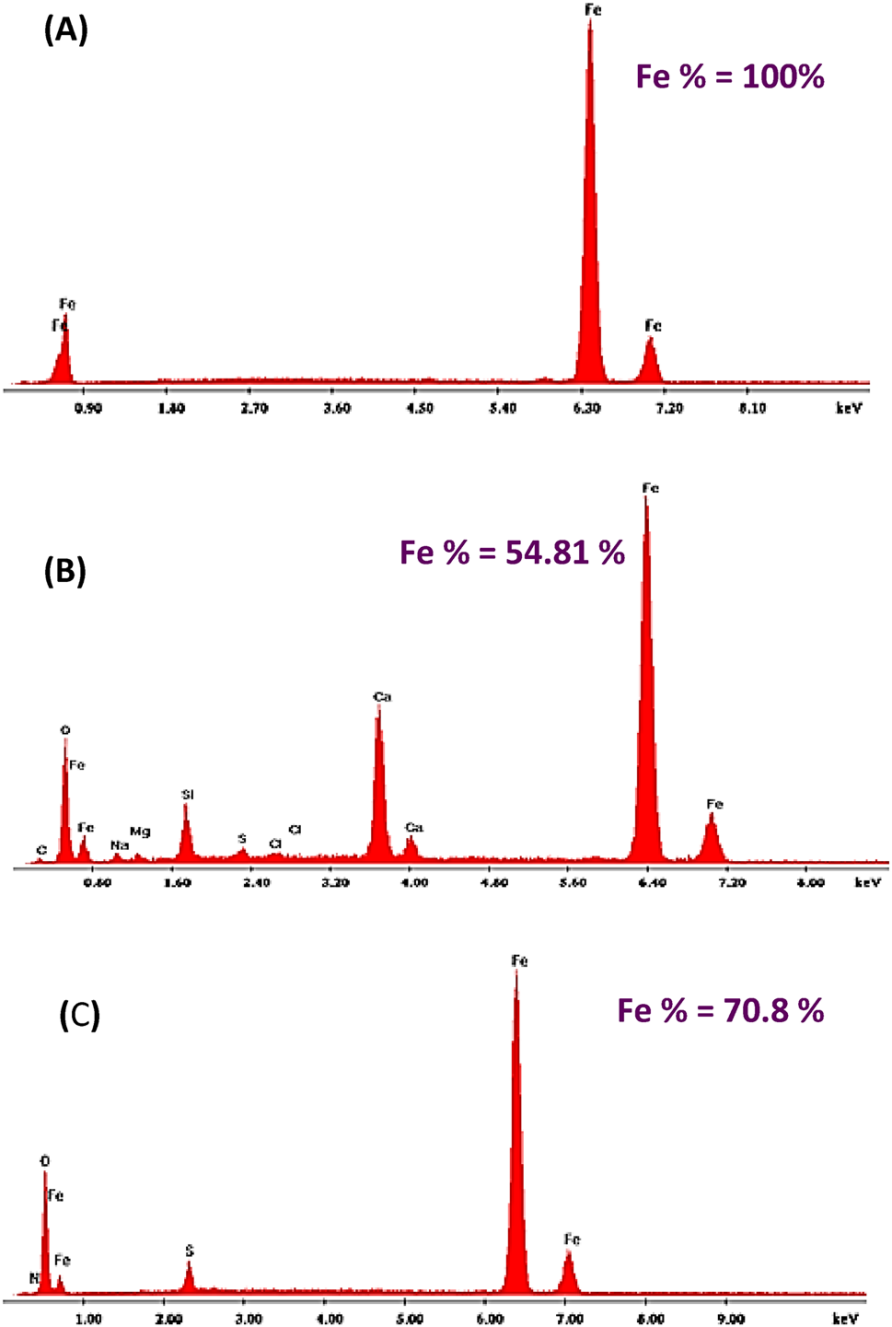
EDX for C steel coupons: (**A**) Before treatment with formation water, (**B**) in Formation water and (**C**) in 2.5% of orange peel extract (OPE).

To investigate the effects of orange peel extract (OPE) on the corrosion process, SEM micrographs of carbon steel exposed to formation water (FW) were examined in Fig. 11. The EDX spectra were utilized to identify the elements present on the carbon steel surface in the presence or absence of OPE (Fig. 12). Energy-dispersive X-ray spectroscopy (EDXS) in combination with scanning electron microscopy (SEM) was employed to determine the chemical composition (%) of the metal surface specimens with and without OPE^61,62^. Table 9 presents the atomic contents (mass%) obtained from the EDX spectrum.

In Fig. 11a, the image of abraded carbon steel revealed transparent and smooth emery paper scratches. Correspondingly, Fig. 12a and Table 9 showed a prominent iron (Fe) peak representing 100% Fe content in its untreated state. Notably, the peaks for carbon (C) and oxygen (O) were not observed, indicating that the spectrum was influenced by Fe^63^. Consequently, the researchers relied on the intensity of the Fe peak to estimate the thickness of the protective layer in the absence and presence of the inhibitor.

Figure 11b depicted severely corroded carbon steel exposed to formation water for a month, showing roughness and voids on the surface. The accompanying EDX spectrum displayed peaks corresponding to elements such as sodium (Na), silicon (Si), calcium (Ca), magnesium (Mg), chlorine (Cl), sulfur (S), and oxygen (O). These elements were attributed to constituents of the saline formation water (e.g., Ca^2+^, Mg^2+^, Clˉ, and SO_4_^2−^) as well as iron oxides, indicating the accumulation of corrosion products on the metal surface. Figure 12b and Table 9 demonstrated a significant decrease in the Fe% content, reaching approximately 54.81% of its original composition, indicating severe damage to the surface.

In contrast, Fig. 11c exhibited less damage in the micrograph when 2.5% orange peel extract (OPE) was present. Additionally, a decrease in the amount of corrosion products was observed in the corresponding EDX spectrum, indicating the formation of a protective film. Figure 12c displayed the appearance of an oxygen (O) signal, indicating the coverage of the inhibitor molecule.

The incorporation of orange peel extract in the system resulted in an increase in the Fe element content to 70.8%, as observed in the EDX patterns (Fig. 12c and Table 9). This increase can be attributed to the formation of a protective layer that effectively shields the surface from corrosion.

The surface examinations conducted confirm the anti-corrosion properties of orange peel extract (OPE) for carbon steel in formation water (FW).

### Inhibition mechanism

In the absence of orange peel extract (OPE), the corrosion of carbon steel (CS) in formation water (FW) is primarily driven by metal dissolution. Anodic sites on the CS surface lead to the release of iron (Fe^2+^) ions, which dissolve in the solution and react with O^2^ and/or (HCO_3_)^−1^ to produce iron oxide and/or iron carbonate corrosion products. The presence of chloride ions weakens the passivation layer, enabling continuous metal dissolution. Simultaneously, electrons generated by metal dissolution migrate to cathodic sites on the metal surface, where they reduce H_2_O to form OH ions^46^.

**Figure 13:**
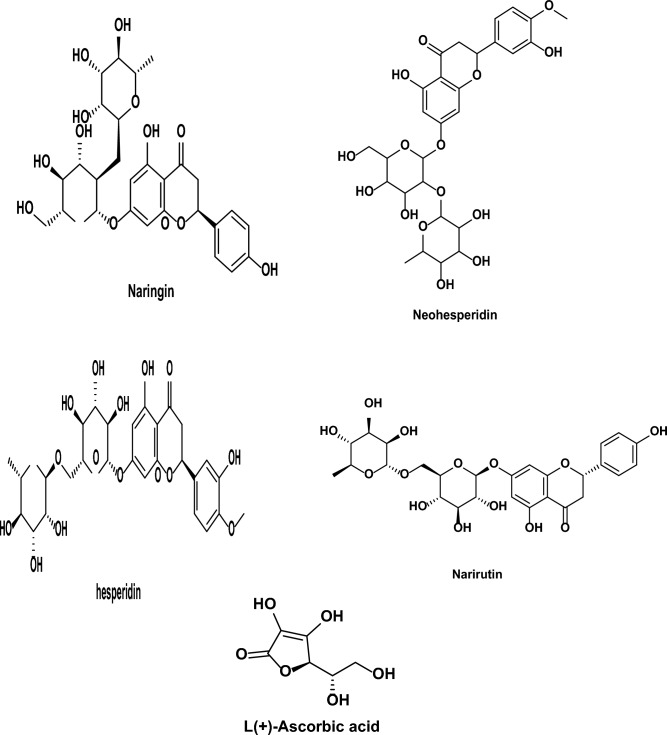
Chemical structures of: (**a**) Naringin, (**b**) Neohesperidin, (**c**) Hesperidin (**d**) Narirutin and (**e**) Ascorbic acid.

**Figure 14:**
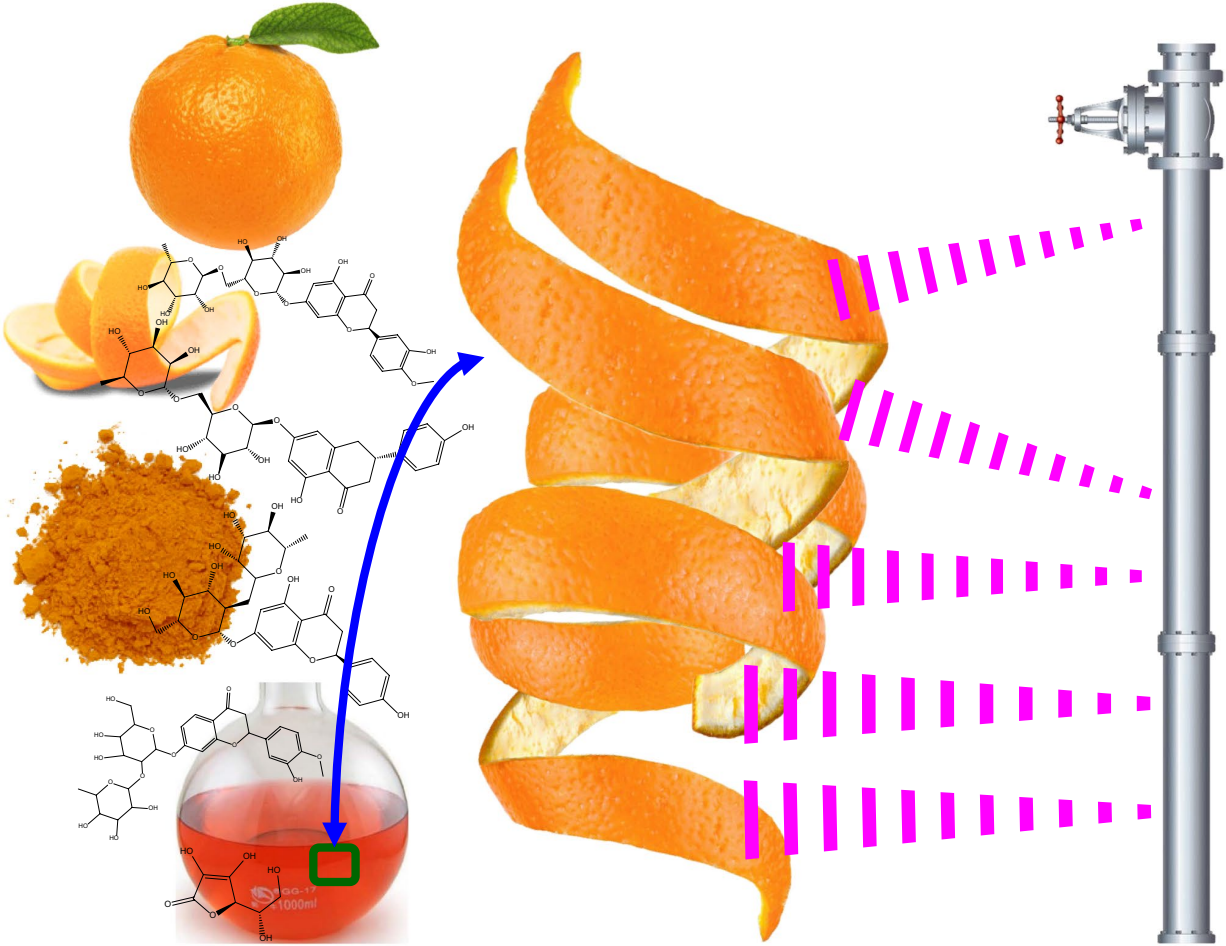
Inhibition mechanism for orange peel extracts on petroleum pipelines.

The corrosion-inhibiting properties of orange peel extract (OPE) can be attributed to its composition, which includes organic compounds like Neohesperidin, Hesperidin, Naringin, Narirutin, and Ascorbic Acid (Fig. 13). These organic molecules possess polar functional groups with oxygen (O) heteroatoms, as well as conjugated double bonds or aromatic rings that serve as primary sites for adsorption. Consequently, the adsorption of OPE on the metal surface leads to the formation of a protective layer, effectively limiting the corrosion of carbon steel (CS) in formation water (FW) (Fig. 14)^64^.

## Conclusions

Orange peel extract (OPE) acts as a mixed-type corrosion inhibitor for carbon steel (CS) in formation water (FW) solutions. Increasing OPE concentrations and lowering temperatures enhance the inhibition efficiency by facilitating the adsorption of OPE components onto the steel surface. The adsorption behavior of OPE compounds conforms to the Langmuir adsorption isotherm.

The use of OPE as a corrosion inhibitor for CS in FW solutions holds promising prospects. The observed improvement in inhibition efficiency with higher OPE concentrations and lower temperatures suggests the potential for optimizing OPE-based inhibitor formulations. Further investigation can focus on identifying the specific compounds in OPE that contribute to its inhibitory effect, enabling the development of targeted corrosion inhibitors. Additionally, studying the adsorption mechanism following the Langmuir adsorption isotherm can provide insights into the binding interactions between OPE compounds and the steel surface. This knowledge can guide the design of more effective and environmentally friendly corrosion inhibitors, leading to reduced maintenance costs and enhanced longevity of equipment and infrastructure.

## Data Availability

The datasets used and/or analyzed during the current study are available from the corresponding author Olfat E. Elazabawy.

## References

[1] Migahed MA, Elgendy A, El-Rabiei MM, Nady H, Zaki EG (2018). Novel Gemini cationic surfactants as anti-corrosion for X-65 steel dissolution in oilfield produced water under sweet conditions: Combined experimental and computational investigations. J. Mol. Struct..

[2] El-Taib Heakal F, Deyab MA, Osman MM, Elkholy AE (2018). Performance of *Centaurea cyanus* aqueous extract towards corrosion mitigation of carbon steel in saline formation water. Desalination.

[3] Srivastava M (2018). Low cost aqueous extract of *Pisum sativum* peels for inhibition of mild steel corrosion. J. Mol. Liq..

[4] Shaban MM (2020). Novel trimeric cationic pyrdinium surfactants as bi-functional corrosion inhibitors and antiscalants for API 5L X70 carbon steel against oilfield formation water. J. Mol. Liq..

[5] Askari M, Aliofkhazraei M, Jafari R, Hamghalam P, Hajizadeh A (2021). Downhole corrosion inhibitors for oil and gas production—A review. Appl. Surf. Sci. Adv..

[6] Chauhan DS, Quraishi MA, Qurashi A (2021). Recent trends in environmentally sustainable Sweet corrosion inhibitors. J. Mol. Liq..

[7] da Rocha JC, da Cunha Ponciano Gomes JA, D’Elia E (2010). Corrosion inhibition of carbon steel in hydrochloric acid solution by fruit peel aqueous extracts. Corros. Sci..

[8] Umoren SA, Eduok UM, Solomon MM, Udoh AP (2016). Corrosion inhibition by leaves and stem extracts of *Sida acuta* for mild steel in 1M H_2_SO_4_ solutions investigated by chemical and spectroscopic techniques. Arab. J. Chem..

[9] Abbas MA, Bedair MA, El-Azabawy OE, Gad ES (2021). Anticorrosion effect of ethoxylate sulfanilamide compounds on carbon steel in 1 M hydrochloric acid: Electrochemical and theoretical studies. ACS Omega.

[10] M’hiri N (2016). Corrosion inhibition of carbon steel in acidic medium by orange peel extract and its main antioxidant compounds. Corros. Sci..

[11] Chidiebere MA, Ogukwe CE, Oguzie KL, Eneh CN, Oguzie EE (2012). Corrosion inhibition and adsorption behavior of *Punica granatum* extract on mild steel in acidic environments: experimental and theoretical studies. Ind. Eng. Chem. Res..

[12] Behpour M, Ghoreishi SM, Khayatkashani M, Soltani N (2012). Green approach to corrosion inhibition of mild steel in two acidic solutions by the extract of *Punica granatum* peel and main constituents. Mater. Chem. Phys..

[13] Loto RT, Loto CA (2020). Inhibition effect of *apium graveolens*, *punica granatum*, and *camellia sinensis* extracts on plain carbon steel. Cogent Eng..

[14] Abboud Y (2016). Corrosion inhibition of carbon steel in hydrochloric acid solution using pomegranate leave extracts. Corros. Eng. Sci. Technol..

[15] Kamal C, Sethuraman MG (2012). Spirulina platensis—A novel green inhibitor for acid corrosion of mild steel. Arab. J. Chem..

[16] Soltani N (2014). Silybum marianum extract as a natural source inhibitor for 304 stainless steel corrosion in 1.0M HCl. J. Ind. Eng. Chem..

[17] Zhang C, Zhao J (2018). Inhibition effects of orange peel extract on the corrosion of Q235 steel in CO_2_-saturated and CO_2_/H_2_S coexistent brine solutions. Res. Chem. Intermed..

[18] de Souza FS, Spinelli A (2009). Caffeic acid as a green corrosion inhibitor for mild steel. Corros. Sci..

[19] Banerjee S, Srivastava V, Singh MM (2012). Chemically modified natural polysaccharide as green corrosion inhibitor for mild steel in acidic medium. Corros. Sci..

[20] Karattu Veedu K, Peringattu Kalarikkal T, Jayakumar N, Gopalan NK (2019). Anticorrosive performance of *Mangifera indica* L. leaf extract-based hybrid coating on steel. ACS Omega.

[21] Ma X (2019). Sunflower head pectin with different molecular weights as promising green corrosion inhibitors of carbon steel in hydrochloric acid solution. ACS Omega.

[22] Saxena A, Prasad D, Haldhar R, Singh G, Kumar A (2018). Use of *Sida cordifolia* extract as green corrosion inhibitor for mild steel in 0.5 M H_2_SO_4_. J. Environ. Chem. Eng..

[23] Pal S (2019). Experimental and theoretical investigation of aqueous and methanolic extracts of *Prunus dulcis* peels as green corrosion inhibitors of mild steel in aggressive chloride media. J. Mol. Liq..

[24] Asfia MP, Rezaei M, Bahlakeh G (2020). Corrosion prevention of AISI 304 stainless steel in hydrochloric acid medium using garlic extract as a green corrosion inhibitor: Electrochemical and theoretical studies. J. Mol. Liq..

[25] Verma C, Ebenso EE, Bahadur I, Quraishi MA (2018). An overview on plant extracts as environmental sustainable and green corrosion inhibitors for metals and alloys in aggressive corrosive media. J. Mol. Liq..

[26] Sowmyashree AS (2023). Potential sustainable electrochemical corrosion inhibition study of *Citrus limetta* on mild steel surface in aggressive acidic media. J. Mater. Res. Technol..

[27] Wu Y (2020). Orange peel extracts as biodegradable corrosion inhibitor for magnesium alloy in NaCl solution: Experimental and theoretical studies. J. Taiwan Inst. Chem. Eng..

[28] Ayodeji A (2022). Corrosion inhibitive performance of the waste orange peels (*Citrus Sinensis*) on A36 mild steel in 1M HCl. Int. J. Electrochem. Sci..

[29] Hassannejad H, Nouri A (2018). Sunflower seed hull extract as a novel green corrosion inhibitor for mild steel in HCl solution. J. Mol. Liq..

[30] Muthukrishnan P, Jeyaprabha B, Prakash P (2017). Adsorption and corrosion inhibiting behavior of *Lannea coromandelica* leaf extract on mild steel corrosion. Arab. J. Chem..

[31] Raghavendra N, Ishwara Bhat J (2019). Application of green products for industrially important materials protection: An amusing anticorrosive behavior of tender arecanut husk (green color) extract at metal-test solution interface. Measurement.

[32] Abd El-raouf M, El-Azabawy OE, El-Azabawy RE (2015). Investigation of adsorption and inhibitive effect of acid red GRE (183) dye on the corrosion of carbon steel in hydrochloric acid media. Egypt. J. Pet..

[33] Al-Sabagh AM, Elsabee M, Elazabawy OE, El-Tabey AE (2010). Corrosion inhibition efficiency of polytriethanolamine surfactants for pipe-lines carbon steel in 1M HCl. J. Dispers. Sci. Technol..

[34] Zhang QH (2021). Two amino acid derivatives as high efficient green inhibitors for the corrosion of carbon steel in CO_2_-saturated formation water. Corros. Sci..

[35] Anaee RA, Tomi IHR, Abdulmajeed MH, Naser SA, Kathem MM (2019). Expired Etoricoxib as a corrosion inhibitor for steel in acidic solution. J. Mol. Liq..

[36] Al-Sabagh AM, Nasser NM, El-Azabawy OE, Tabey AEE (2016). Corrosion inhibition behavior of new synthesized nonionic surfactants based on amino acid on carbon steel in acid media. J. Mol. Liq..

[37] Kumar H, Yadav V, Saha SK, Kang N (2021). Adsorption and inhibition mechanism of efficient and environment friendly corrosion inhibitor for mild steel: Experimental and theoretical study. J. Mol. Liq..

[38] Baran E, Cakir A, Yazici B (2019). Inhibitory effect of *Gentiana olivieri *extracts on the corrosion of mild steel in 0.5M HCl: Electrochemical and phytochemical evaluation. Arab. J. Chem..

[39] Abboud Y (2009). Corrosion inhibition of carbon steel in acidic media by *Bifurcaria bifurcata* extract. Chem. Eng. Commun..

[40] Al-Sabagh AM, Kandile NG, Nasser NM, El-Azabawy OE, El-Tabey AE (2015). Investigation of electro and quantum chemical properties of some novel cationic surfactants based on 1,3,5-triethanolhexahydro-1,3,5-triazine as corrosion inhibitors for carbon steel in hydrochloric acid. Chem. Eng. Commun..

[41] Chen T, Chen M, Chen Z, Fu C (2021). Comprehensive investigation of modified polyethyleneimine as an efficient polymeric corrosion inhibitor in neutral medium: Synthesis, experimental and theoretical assessments. J. Mol. Liq..

[42] Chen Z (2021). Green synthesis of corrosion inhibitor with biomass platform molecule: Gravimetrical, electrochemical, morphological, and theoretical investigations. J. Mol. Liq..

[43] Abbout S (2021). Gravimetric, electrochemical and theoretical study, and surface analysis of novel epoxy resin as corrosion inhibitor of carbon steel in 0.5 M H_2_SO_4_ solution. J. Mol. Struct..

[44] Fawzy A, Abdallah M, Zaafarany IA, Ahmed SA, Althagafi II (2018). Thermodynamic, kinetic and mechanistic approach to the corrosion inhibition of carbon steel by new synthesized amino acids-based surfactants as green inhibitors in neutral and alkaline aqueous media. J. Mol. Liq..

[45] Rajan JP, Shrivastava R, Mishra RK (2017). Corrosion inhibition effect of *Clerodendron Colebrookianum Walp* leaves (Phuinam) extract on the acid corrosion of mild steel. Prot. Met. Phys. Chem. Surfaces.

[46] Deyab MA (2016). Inhibition activity of Seaweed extract for mild carbon steel corrosion in saline formation water. Desalination.

[47] Saraswat V, Yadav M (2021). Improved corrosion resistant performance of mild steel under acid environment by novel carbon dots as green corrosion inhibitor. Colloids Surfaces A Physicochem. Eng. Asp..

[48] Fouda AS, Heakal FE, Radwan MS (2009). Role of some thiadiazole derivatives as inhibitors for the corrosion of C-steel in 1 M H_2_SO_4_. J. Appl. Electrochem..

[49] Deyab MA, Mohsen Q, Guo L (2022). Aesculus hippocastanum seeds extract as eco-friendly corrosion inhibitor for desalination plants: Experimental and theoretical studies. J. Mol. Liq..

[50] Abdul Rahiman AFS, Sethumanickam S (2017). Corrosion inhibition, adsorption and thermodynamic properties of poly(vinyl alcohol-cysteine) in molar HCl. Arab. J. Chem..

[51] Deyab MA (2018). Corrosion inhibition of heat exchanger tubing material (titanium) in MSF desalination plants in acid cleaning solution using aromatic nitro compounds. Desalination.

[52] Al-Sabagh AM, Abd-El-Bary HM, El-Ghazawy RA, Mishrif MR, Hussein BM (2012). Corrosion inhibition efficiency of heavy alkyl benzene derivatives for carbon steel pipelines in 1M HCl. Egypt. J. Pet..

[53] Hegazy MA, Samy RM, Labena A, Wadaan MAM, Hozzein WN (2020). 4,4′-(((1E,5E)-pentane-1,5-diylidene)bis(azanylylidene))bis(1-dodecylpyridin-1-ium) bromide as a novel corrosion inhibitor in an acidic solution (part I). Mater. Sci. Eng. C.

[54] Shahmoradi AR (2021). Theoretical and surface/electrochemical investigations of walnut fruit green husk extract as effective inhibitor for mild-steel corrosion in 1M HCl electrolyte. J. Mol. Liq..

[55] Ugin Inbaraj N, Venkatesa Prabhu G (2018). Corrosion inhibition properties of paracetamol based benzoxazine on HCS and Al surfaces in 1M HCl. Prog. Org. Coatings.

[56] Rathod MR, Rajappa SK, Praveen BM, Bharath DK (2021). Investigation of Dolichandra unguis-cati leaves extract as a corrosion inhibitor for mild steel in acid medium. Curr. Res. Green Sustain. Chem..

[57] El-Tabei AS, Hegazy MA, Bedair AH, El Basiony N, Sadeq MA (2021). Experimental and theoretical (DFT&MC) studies for newly synthesized cationic amphiphilic substance based on a naphthol moiety as corrosion inhibitor for carbon steel during the pickling process. J. Mol. Liq..

[58] Begum AA (2021). *Spilanthes acmella* leaves extract for corrosion inhibition in acid medium. Coatings.

[59] Arukalam IO (2014). Durability and synergistic effects of KI on the acid corrosion inhibition of mild steel by hydroxypropyl methylcellulose. Carbohydr. Polym..

[60] Zapata B, Balmaseda J, Fregoso-Israel E, Torres-García E (2009). Thermo-kinetics study of orange peel in air. J. Therm. Anal. Calorim..

[61] Ech-chihbi E (2020). Computational, MD simulation, SEM/EDX and experimental studies for understanding adsorption of benzimidazole derivatives as corrosion inhibitors in 1.0 M HCl solution. J. Alloys Compd..

[62] Goyal M (2020). Isopentyltriphenylphosphonium bromideionic liquid as a newly effective corrosion inhibitor on metal-electrolyte interface in acidic medium: Experimental, surface morphological (SEM-EDX & AFM) and computational analysis. J. Mol. Liq..

[63] Migahed MA (2011). Synthesis of a new family of Schiff base nonionic surfactants and evaluation of their corrosion inhibition effect on X-65 type tubing steel in deep oil wells formation water. Mater. Chem. Phys..

[64] Deyab MA (2016). Experimental evaluation of new inorganic phosphites as corrosion inhibitors for carbon steel in saline water from oil source wells. Desalination.

